# Supplementary material for: arrayMap: A Reference Resource for Genomic Copy Number Imbalances in Human Malignancies
Source: PLoS One. 2012 May 18;7(5):e36944. doi: 10.1371/journal.pone.0036944 (PMC3356349; doi:10.1371/journal.pone.0036944)
Supplement: Figure S1 — Array data sets visualization. Original plots and optimized parameters for GSE21530 which contains 8 intimal sarcoma samples hybridized on Agilent CGH Microarray 244A platform. The normalized probe signal log2 ratios and post-thresholding segmentation results for each array are intuitively displayed. Genomic alterations are represented by horizontal green (gain) and red (loss) lines. Alterations defined here as regions with log2 ratio >0.15 or <−0.15. Simplified schemas of CNAs link to UCSC genome browser for further review. (PDF) [file pone.0036944.s001.pdf]

arrayMap

Search Public Series
Search Public Arrays
Plot Array Data
Progenetix

University of Zurich

News
Citation
FAQ & Guide
Contributing Authors
CompBio Zurich
Atlas of Cytogenetics in Hematology/Oncology
NCI SKY/M-FISH and CGH Database
NCI Cancer Chromosomes

FOLLOW US ON twitter

Share

BY-NC-ND

130.60.44.248
130.60.93.222

Visualization of genomic array data sets

SERIES	GSE21530	[?]
ARRAY ID		[?]
	Show original plots & Load defaults (if existing)	
CASE DATA PLOTTING	no plot refresh	[?]
GOLDEN PATH EDITION	HG18 / 36	[?]
PLOT REGION		[?]
CHROMOSOMES TO PLOT	1:22	[?]
LOSS/GAIN THRESHOLDS	-0.15 – 0.15 Y AXIS FACTOR 1.2	[?]
REGION SIZE	0 – 250000 MIN. PROBES 2	[?]
EMAIL / PASSWORD	your registered email / your received password	[?]
LICENSE AGREEMENT	I agree to license, disclaimer & citation note	
	Filter / replot array data	

8 will be processed.

GSM537760	360 segments
GSM537761	219 segments
GSM537762	177 segments
GSM537763	93 segments
GSM537764	1178 segments
GSM537765	392 segments
GSM537766	69 segments
GSM537767	341 segments

8 arrays were found.

parsing database ...

GSM537760

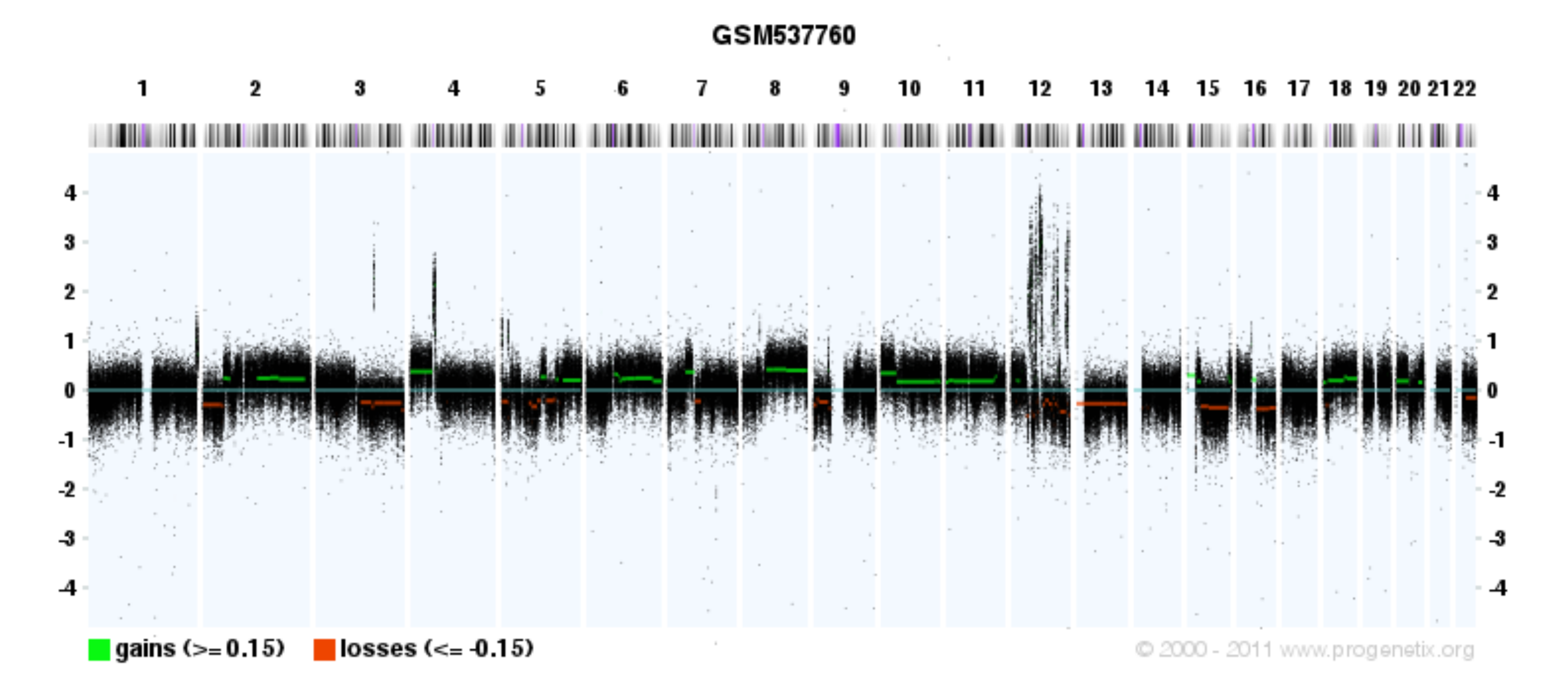

ARRAYPLOT PNG SVG

GSM537761

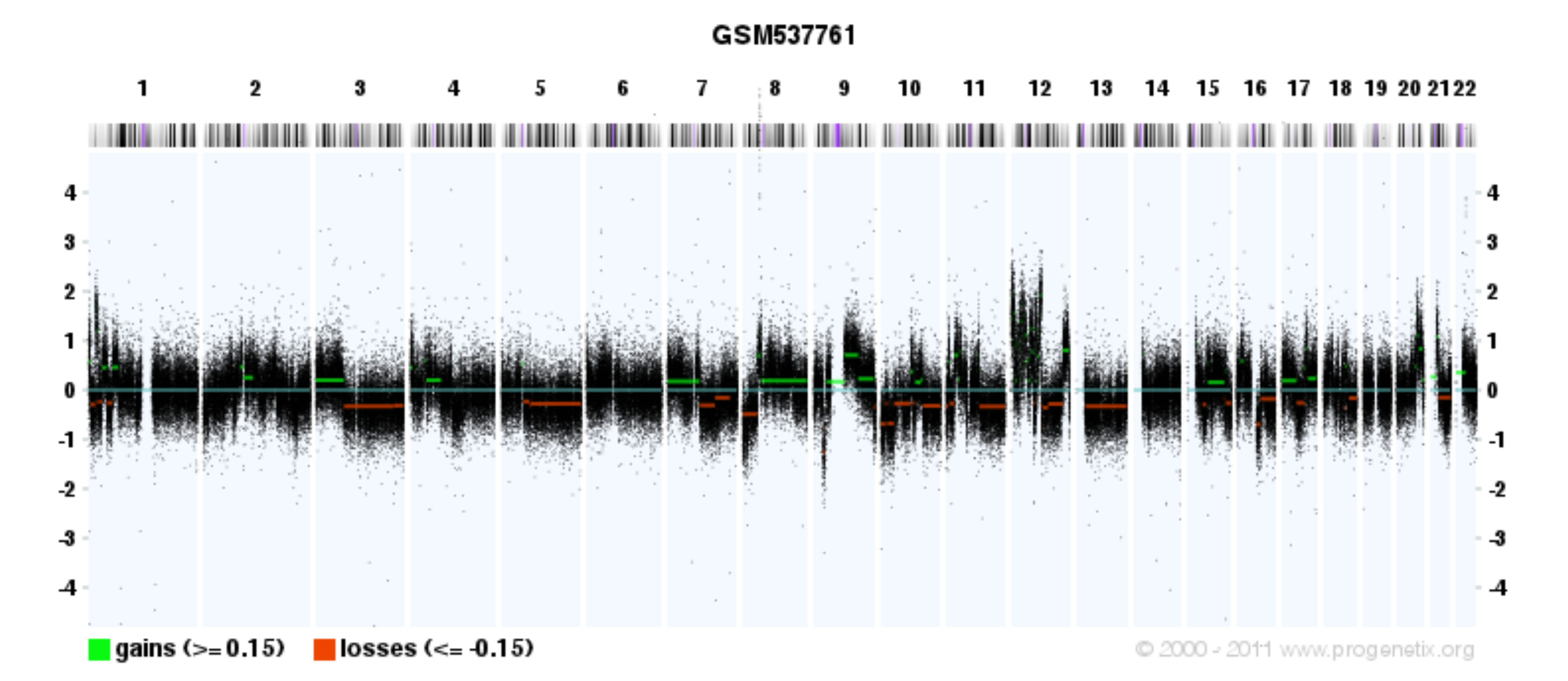

ARRAYPLOT PNG SVG

GSM537762

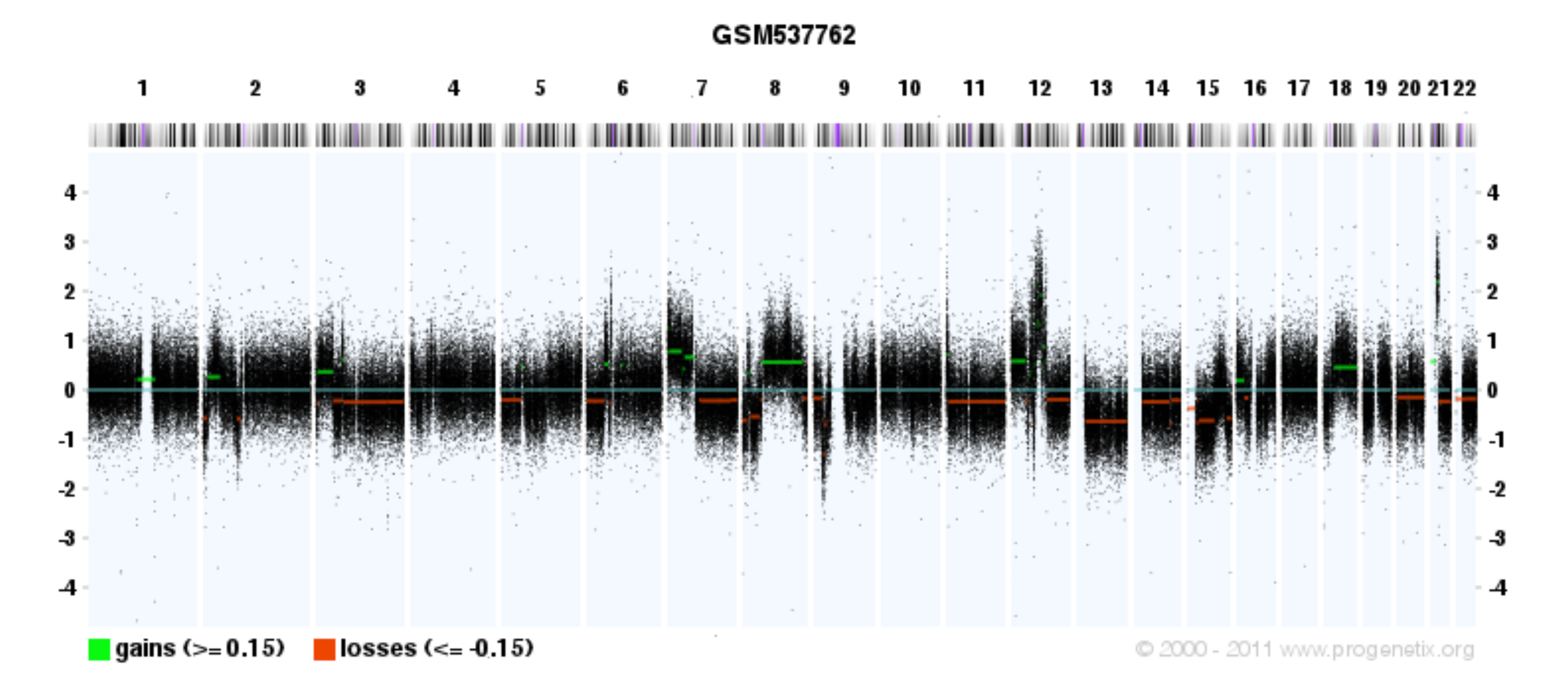

ARRAYPLOT PNG SVG

GSM537763

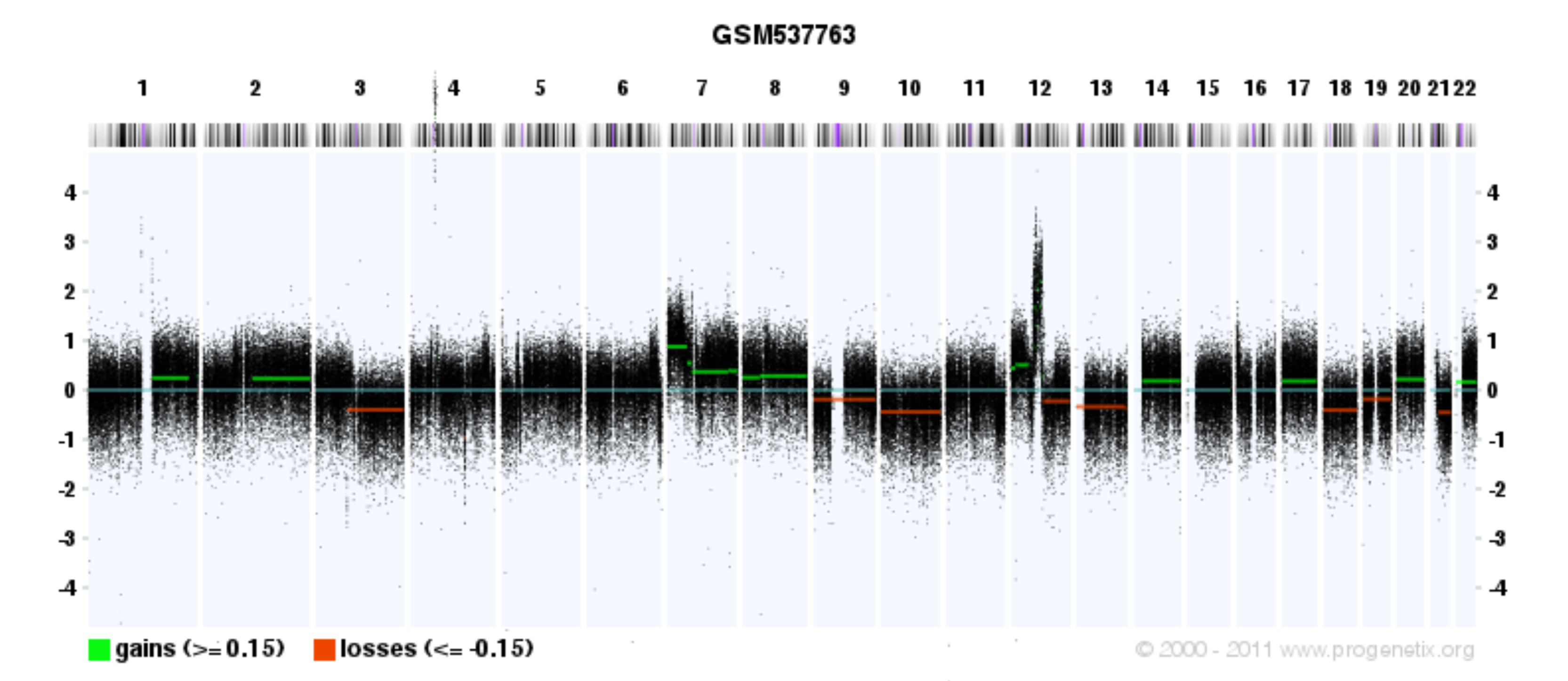

ARRAYPLOT PNG SVG

GSM537764

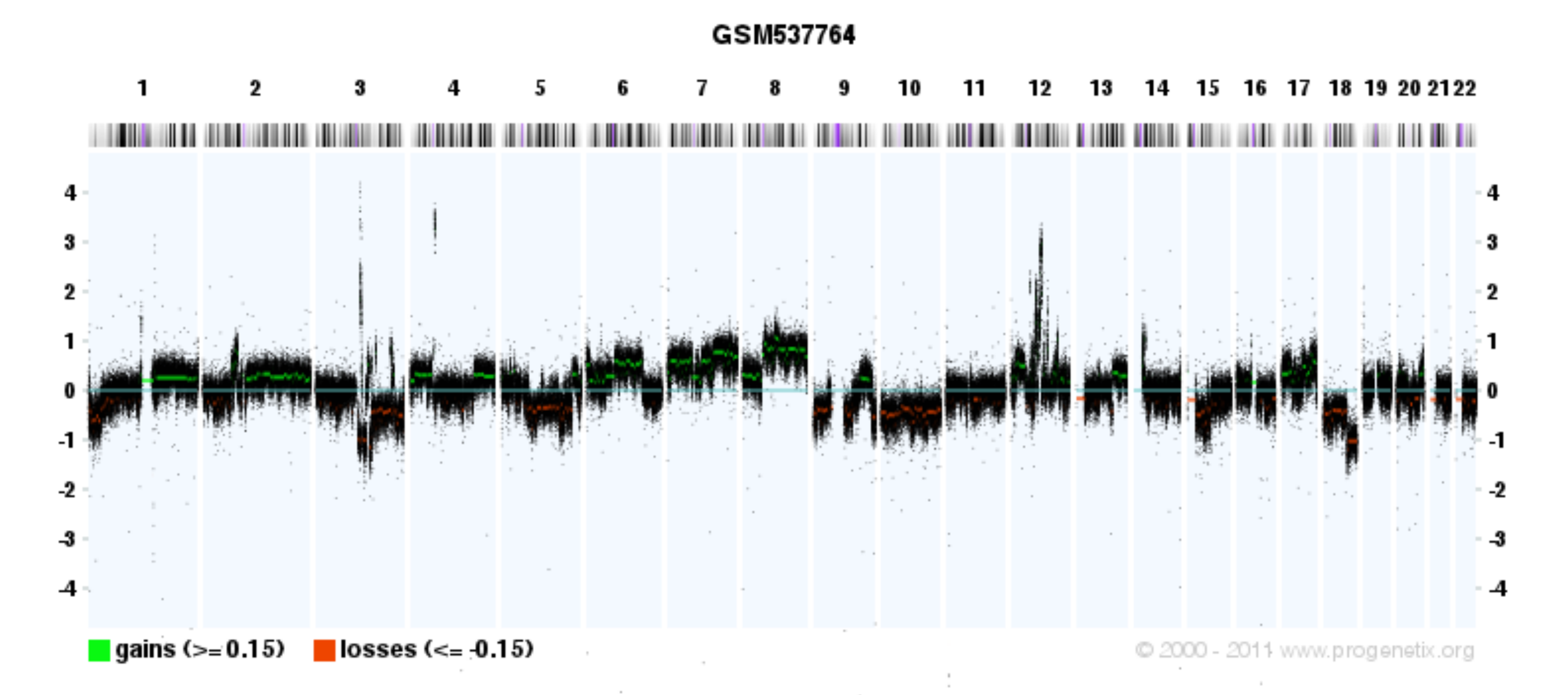

ARRAYPLOT PNG SVG

GSM537765

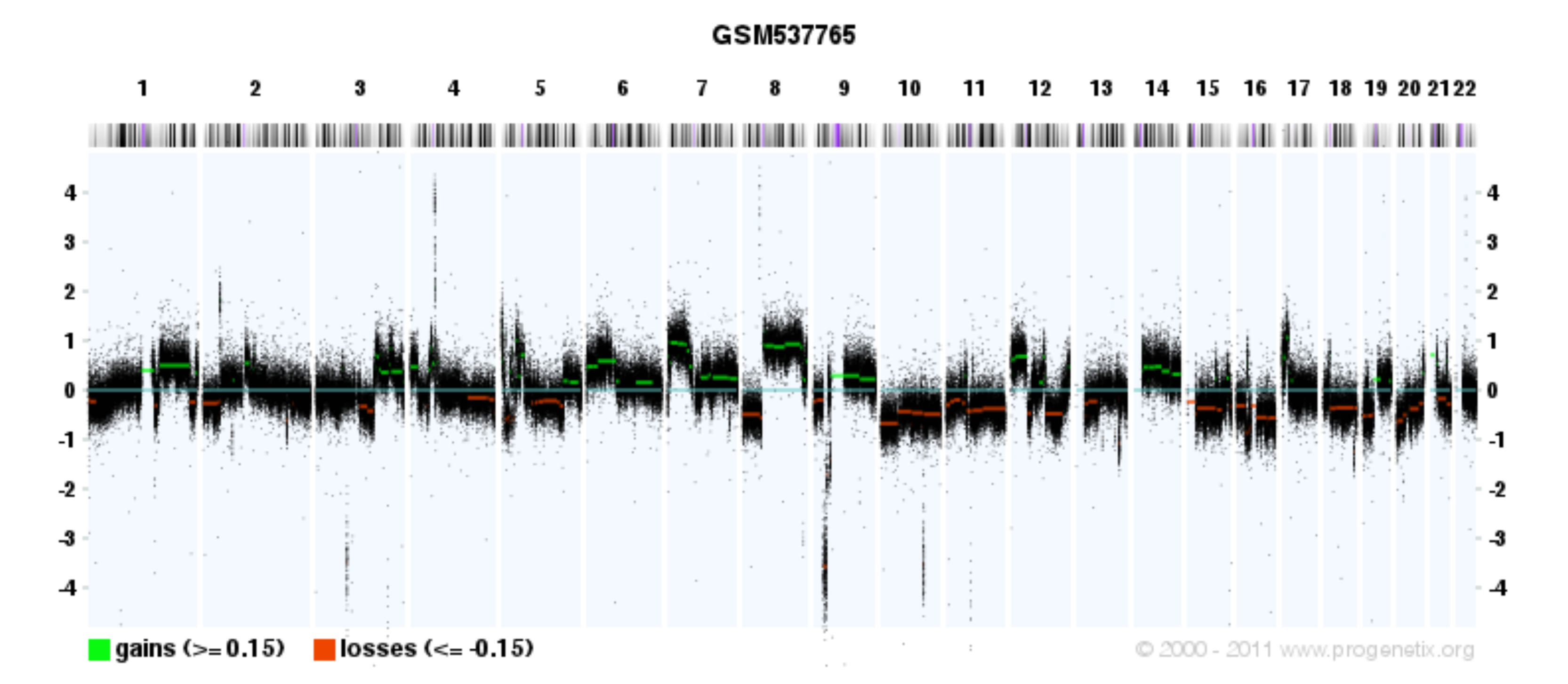

ARRAYPLOT PNG SVG

GSM537766

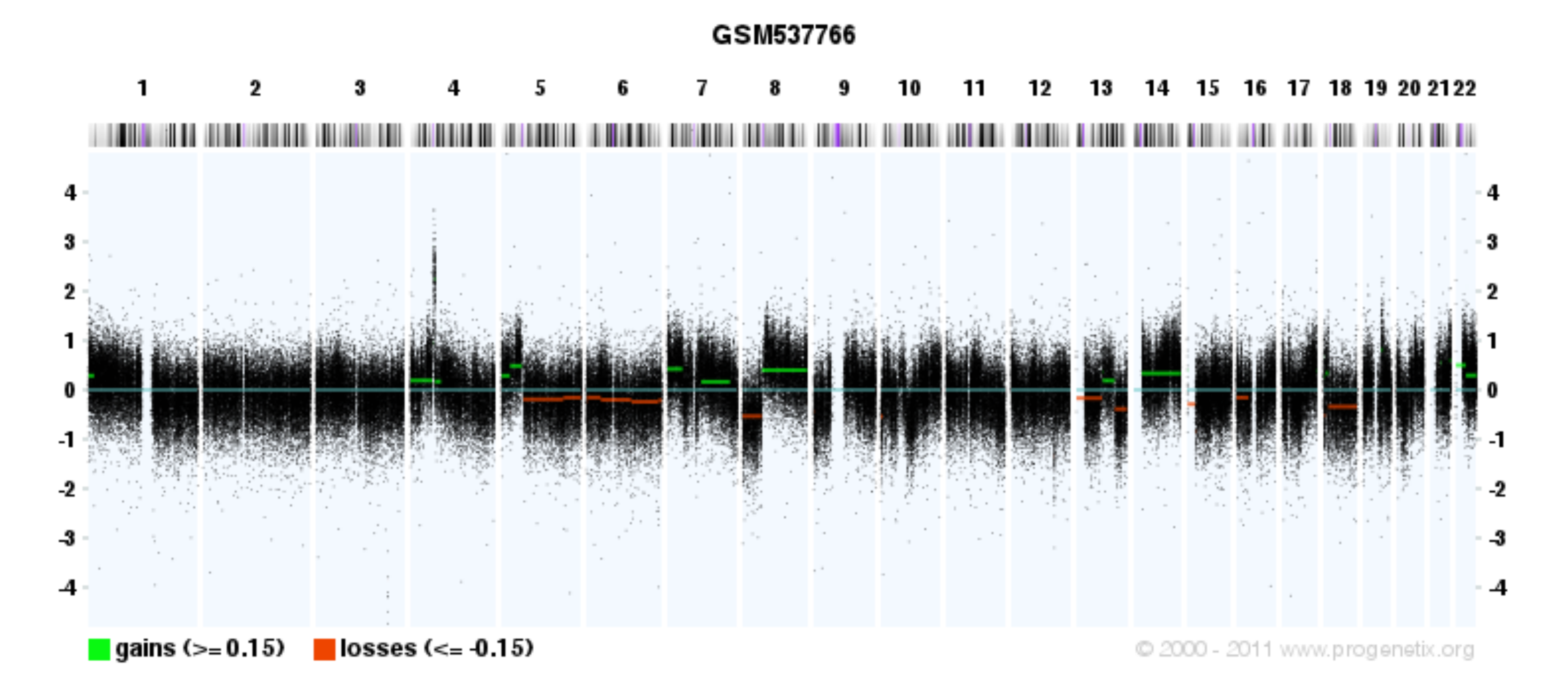

ARRAYPLOT PNG SVG

GSM537767

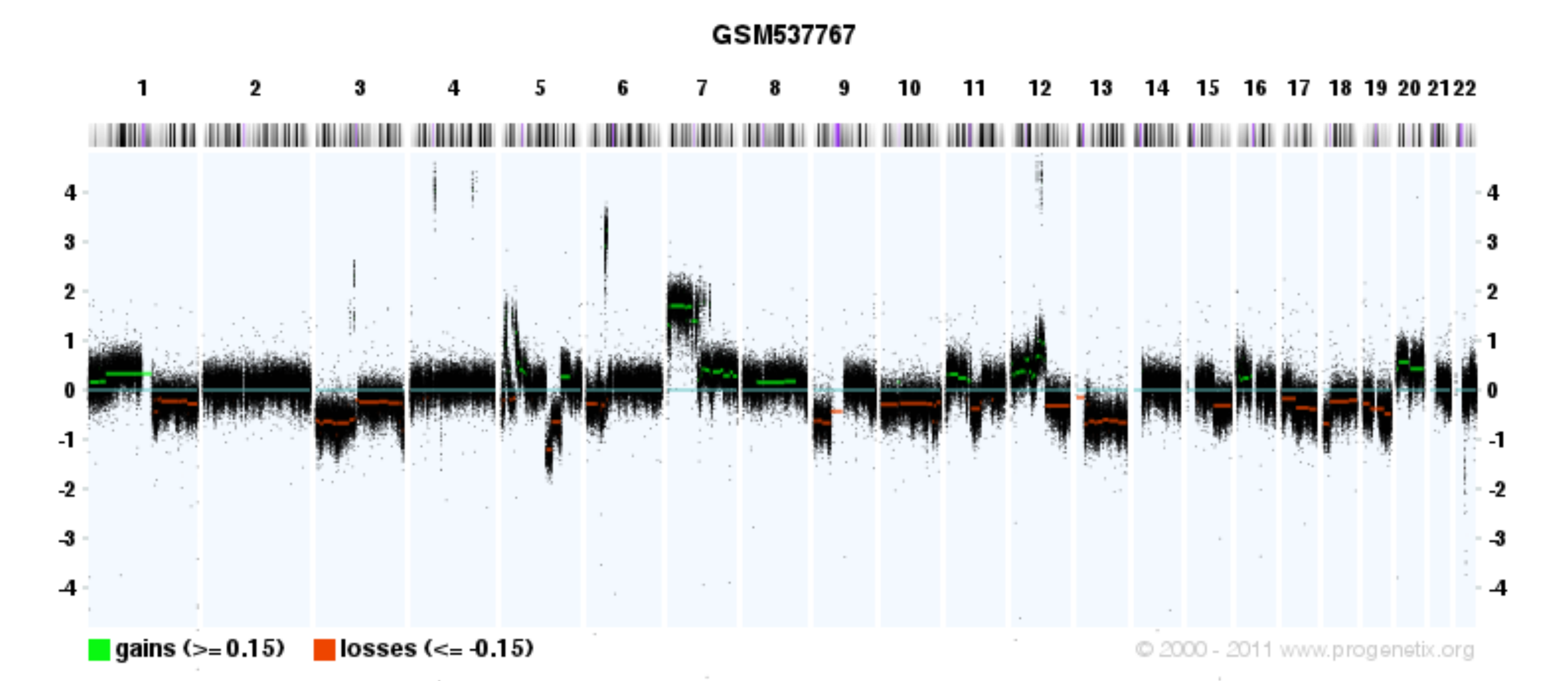

ARRAYPLOT PNG SVG

SELECT ALL [?]

Visualize Data Summary
