# Supplementary material for: arrayMap: A Reference Resource for Genomic Copy Number Imbalances in Human Malignancies
Source: PLoS One. 2012 May 18;7(5):e36944. doi: 10.1371/journal.pone.0036944 (PMC3356349; doi:10.1371/journal.pone.0036944)
Supplement: Figure S3 — Plot single genomic region. In the “Plot Array Data” interface, input the precise location (chr5∶1100000-1400000) in “Plot Region” field. Plots with this region were generated for all 8 arrays in the current series (GSE21530). In this region, there are 5 genes which are shown schematically as colored boxes. CNA status and copy number transition points for these genes are displayed. (PDF) [file pone.0036944.s003.pdf]

arrayMap

- Search Public Series
- Search Public Arrays
- Plot Array Data
- Progenetix

University of Zurich

- News
- Citation
- FAQ & Guide
- Contributing Authors
- CompBio Zurich
- Atlas of Cytogenetics in Hematology/Oncology
- NCI SKY/M-FISH and CGH Database
- NCI Cancer Chromosomes

FOLLOW US ON twitter

Share

(cc) BY-NC-ND

130.60.44.246
89.206.100.176

Visualization of genomic array data sets

SERIES	GSE21530	[?]
ARRAY ID	GSM537760::GSM537762::GSM537765::GSM537763::GSM537767::GSM5	[?]
	Show original plots & Load defaults (if existing)	

CASE DATA PLOTTING	plot raw data values & segments				[?]
GOLDEN PATH EDITION	HG18 / 36				[?]
PLOT REGION	chr5:1100000-1400000				[?]
CHROMOSOMES TO PLOT	1:22				[?]
LOSS/GAIN THRESHOLDS	-0.15	0.15	Y AXIS FACTOR	1.2	[?]
REGION SIZE	0	250000	MIN. PROBES	2	[?]

EMAIL / PASSWORD	your registered email	your received password	[?]
LICENSE AGREEMENT	I agree to license, disclaimer & citation note ☑		
	Filter / replot array data		

8 will be processed.

GSM537760	26 segments
GSM537761	7 segments
GSM537762	4 segments
GSM537763	1 segments
GSM537764	86 segments
GSM537765	63 segments
GSM537766	8 segments
GSM537767	50 segments

☑ GSM537760	Replotted array GSM537760 (1 of 8).
☑ GSM537761	Replotted array GSM537761 (2 of 8).
☑ GSM537762	Replotted array GSM537762 (3 of 8).
☑ GSM537763	Replotted array GSM537763 (4 of 8).
☑ GSM537764	Replotted array GSM537764 (5 of 8).
☑ GSM537765	Replotted array GSM537765 (6 of 8).
☑ GSM537766	Replotted array GSM537766 (7 of 8).
☑ GSM537767	Replotted array GSM537767 (8 of 8).

REPLACE DEFAULT PLOTS	internal use only ...	☑ save plots ☑ save defaults	[?]
	Update plots		

8 arrays were found.

parsing database ...

- ☐ GSM537760
- ☐ GSM537761
- ☐ GSM537762
- ☐ GSM537763
- ☐ GSM537764
- ☐ GSM537765
- ☐ GSM537766
- ☐ GSM537767

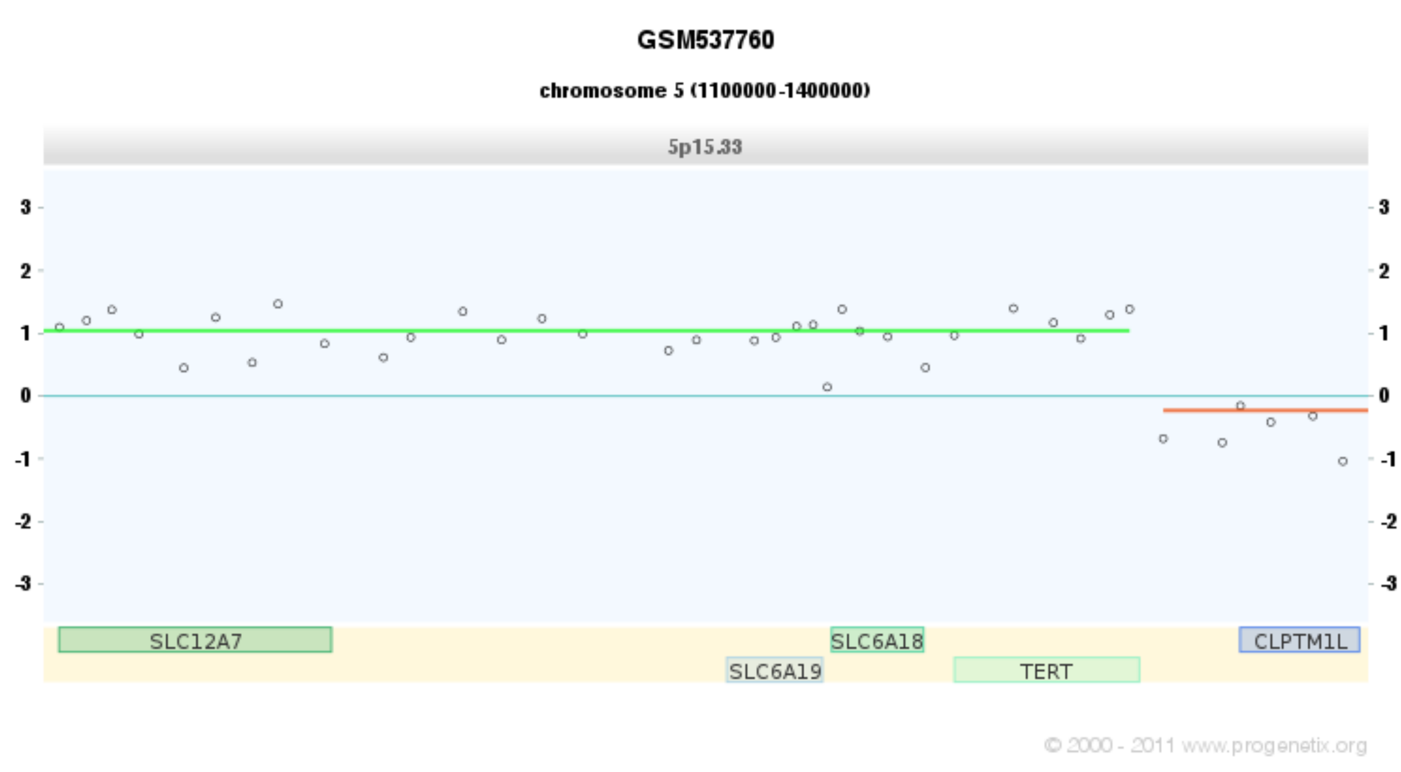

ARRAYPLOT,CHR5,1100000-1400000 PNG SVG

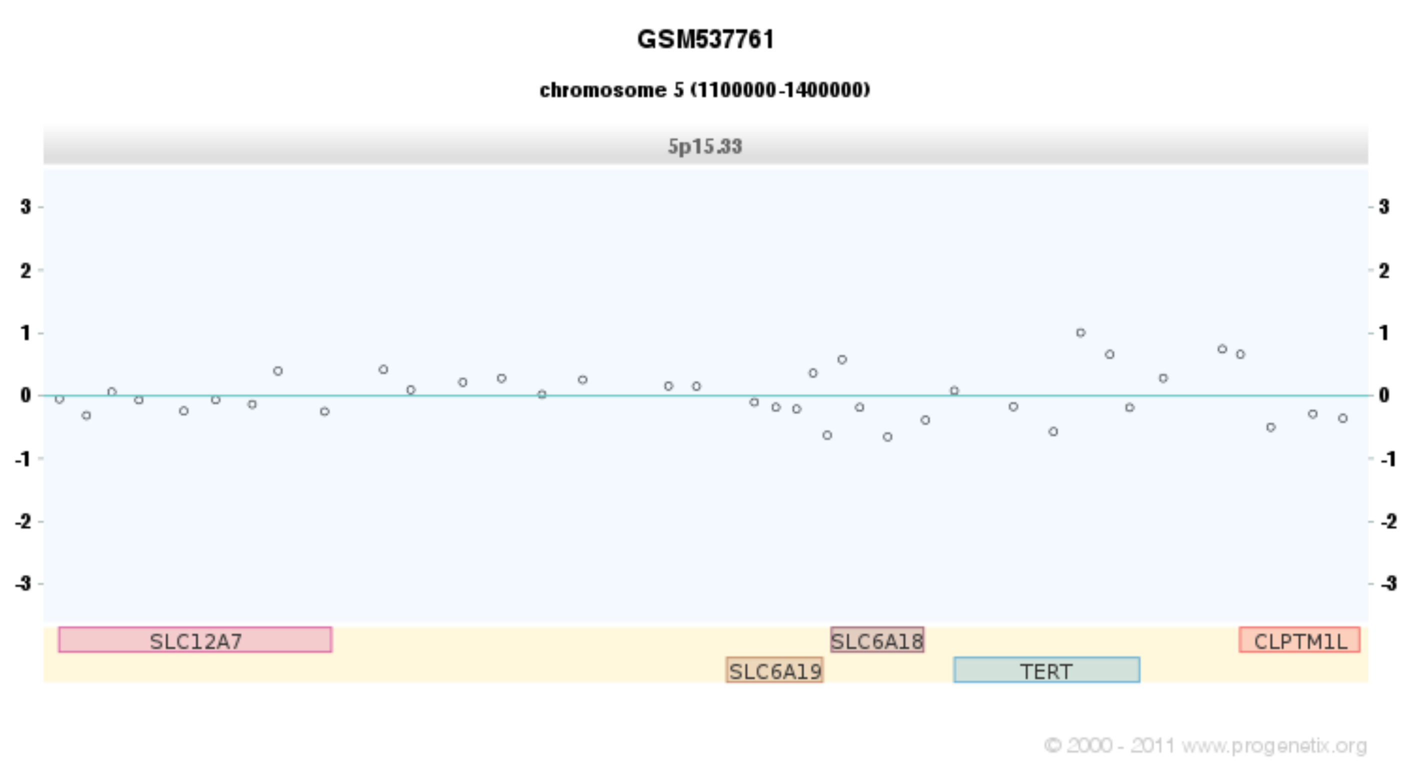

ARRAYPLOT,CHR5,1100000-1400000 PNG SVG

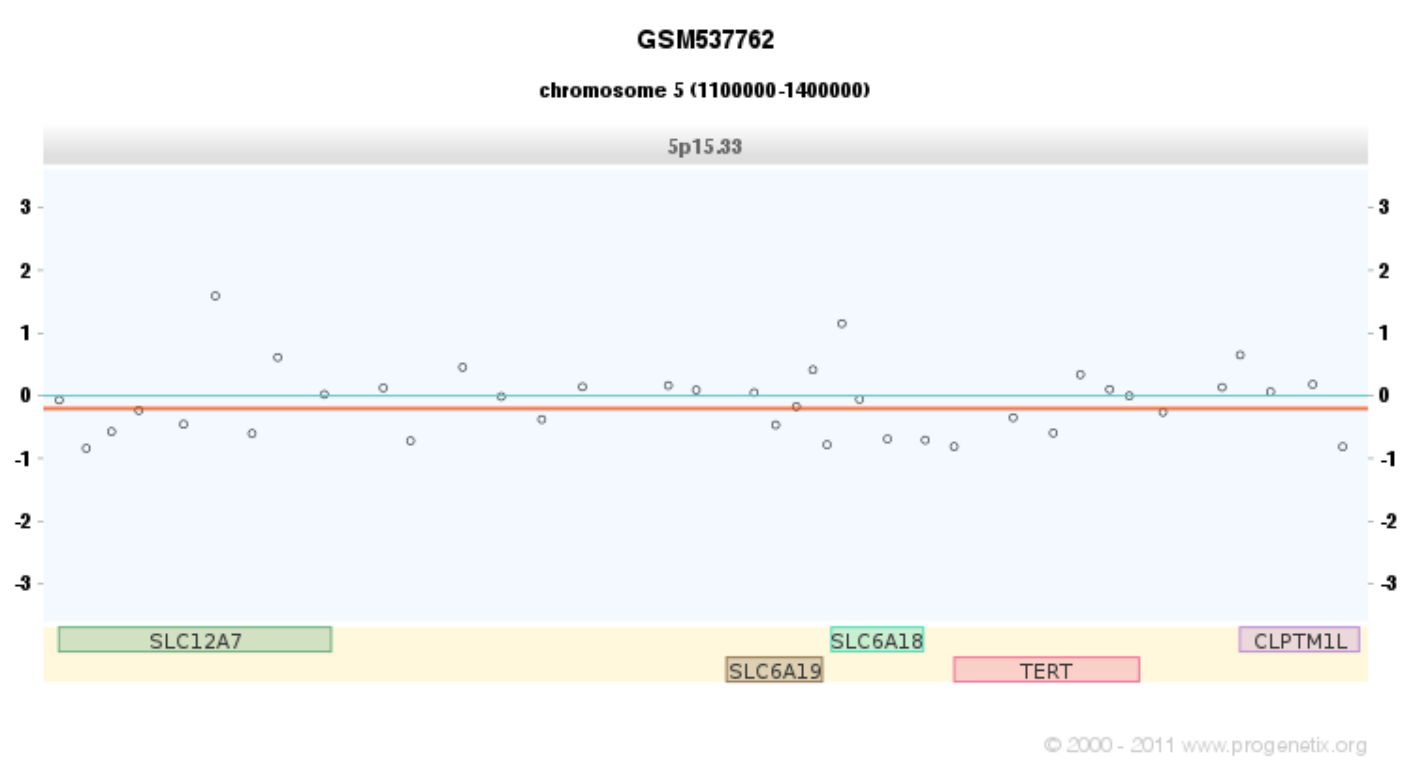

ARRAYPLOT,CHR5,1100000-1400000 PNG SVG

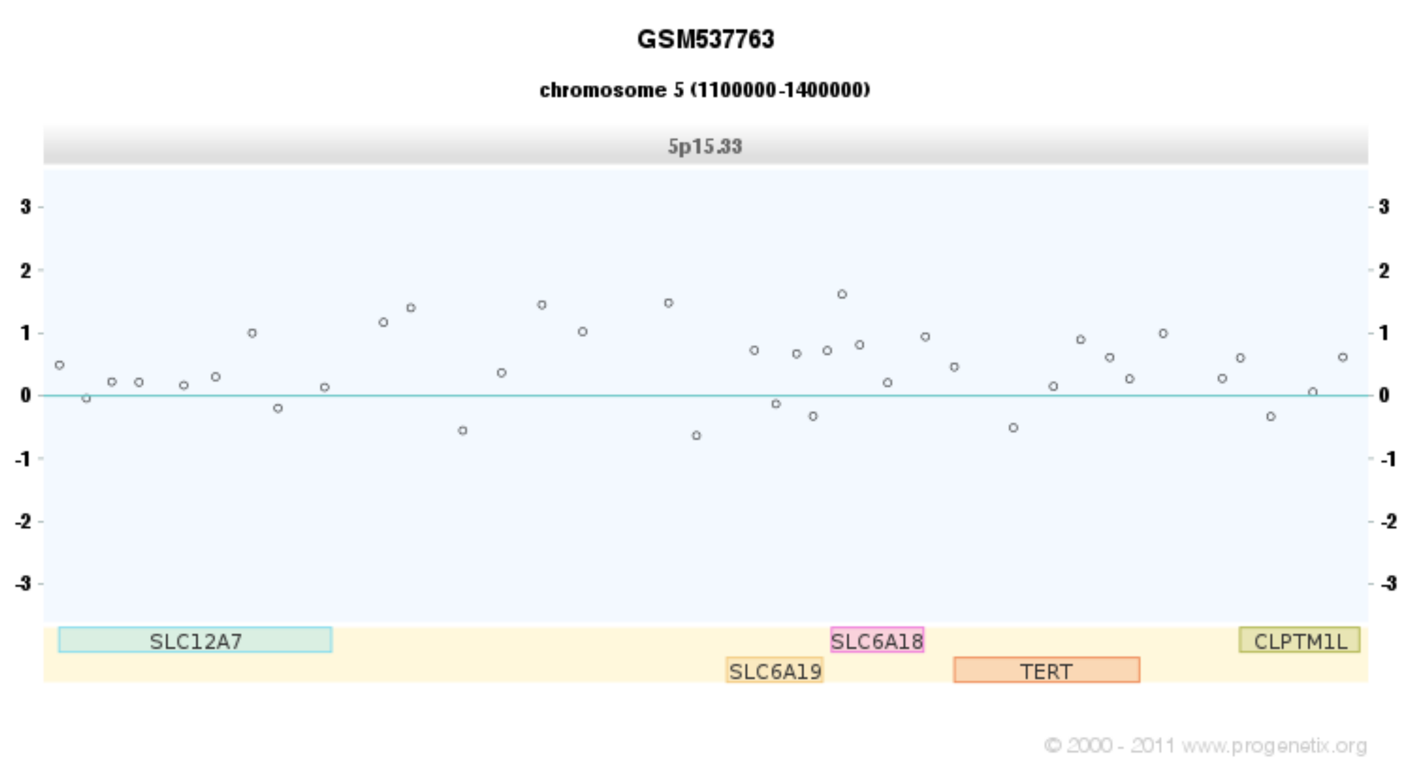

ARRAYPLOT,CHR5,1100000-1400000 PNG SVG

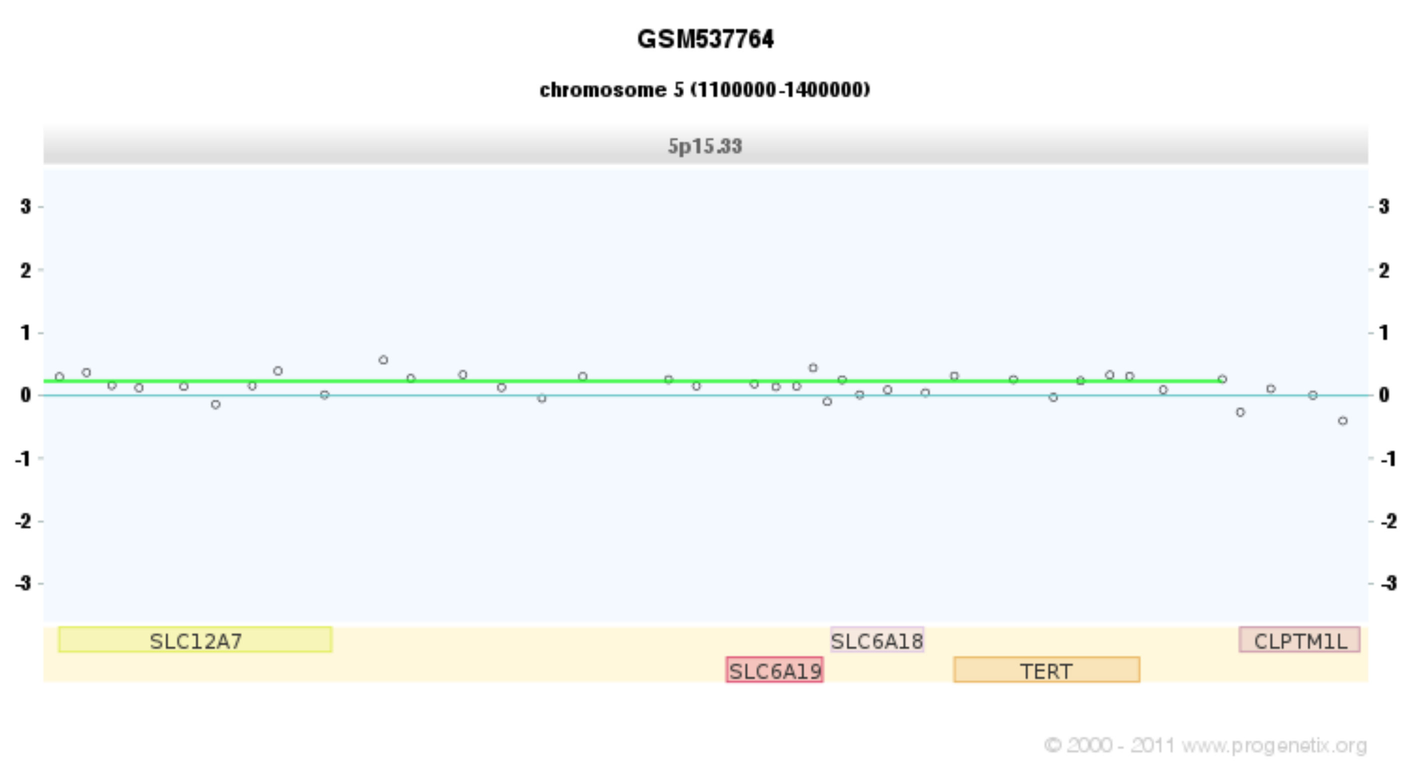

ARRAYPLOT,CHR5,1100000-1400000 PNG SVG

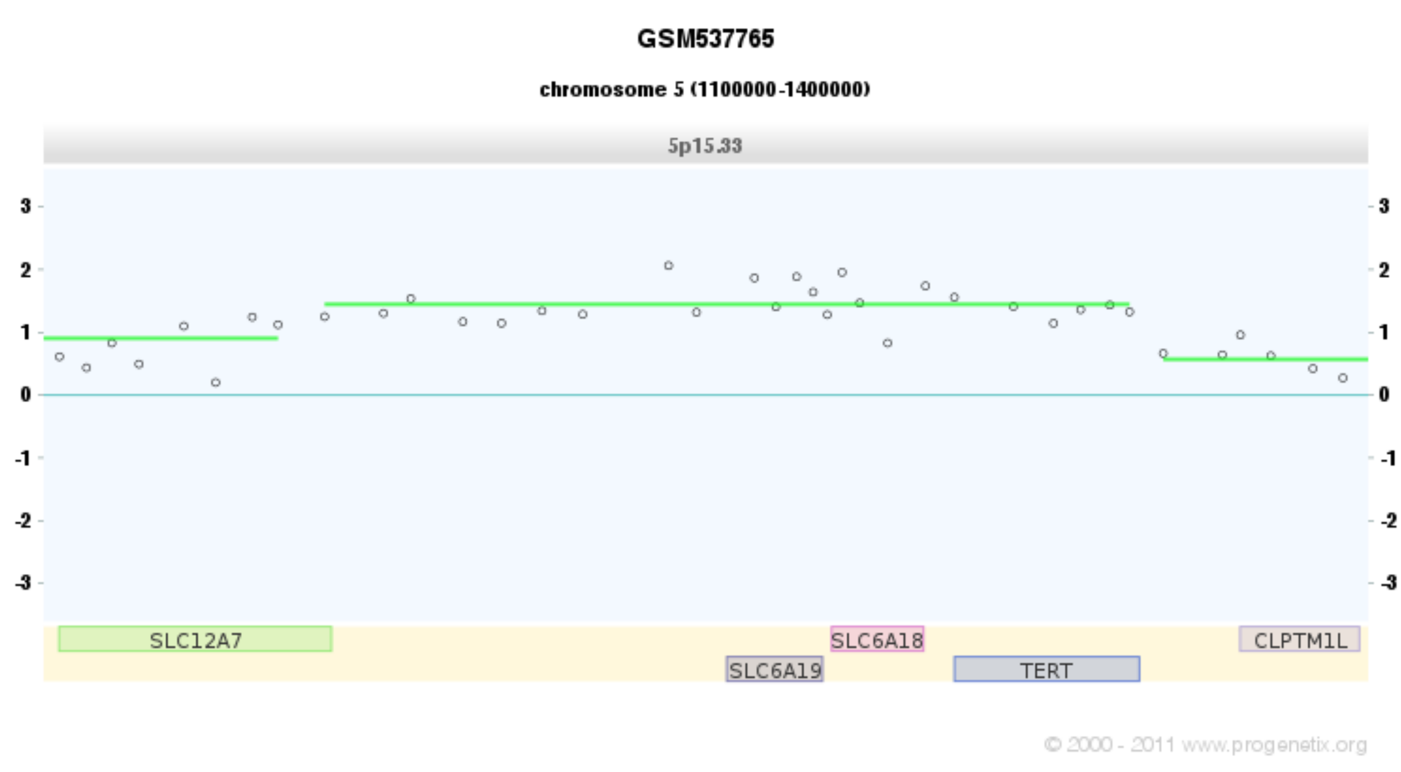

ARRAYPLOT,CHR5,1100000-1400000 PNG SVG

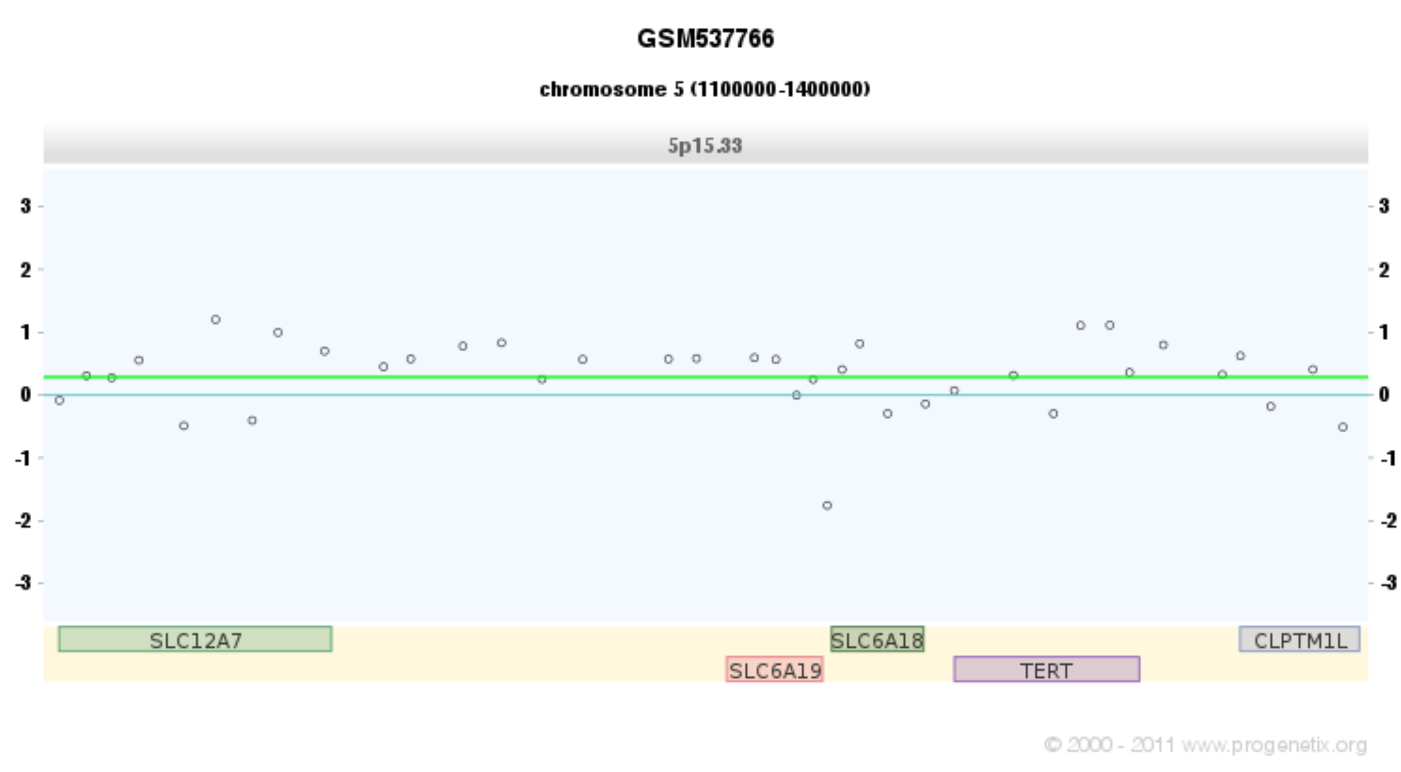

ARRAYPLOT,CHR5,1100000-1400000 PNG SVG

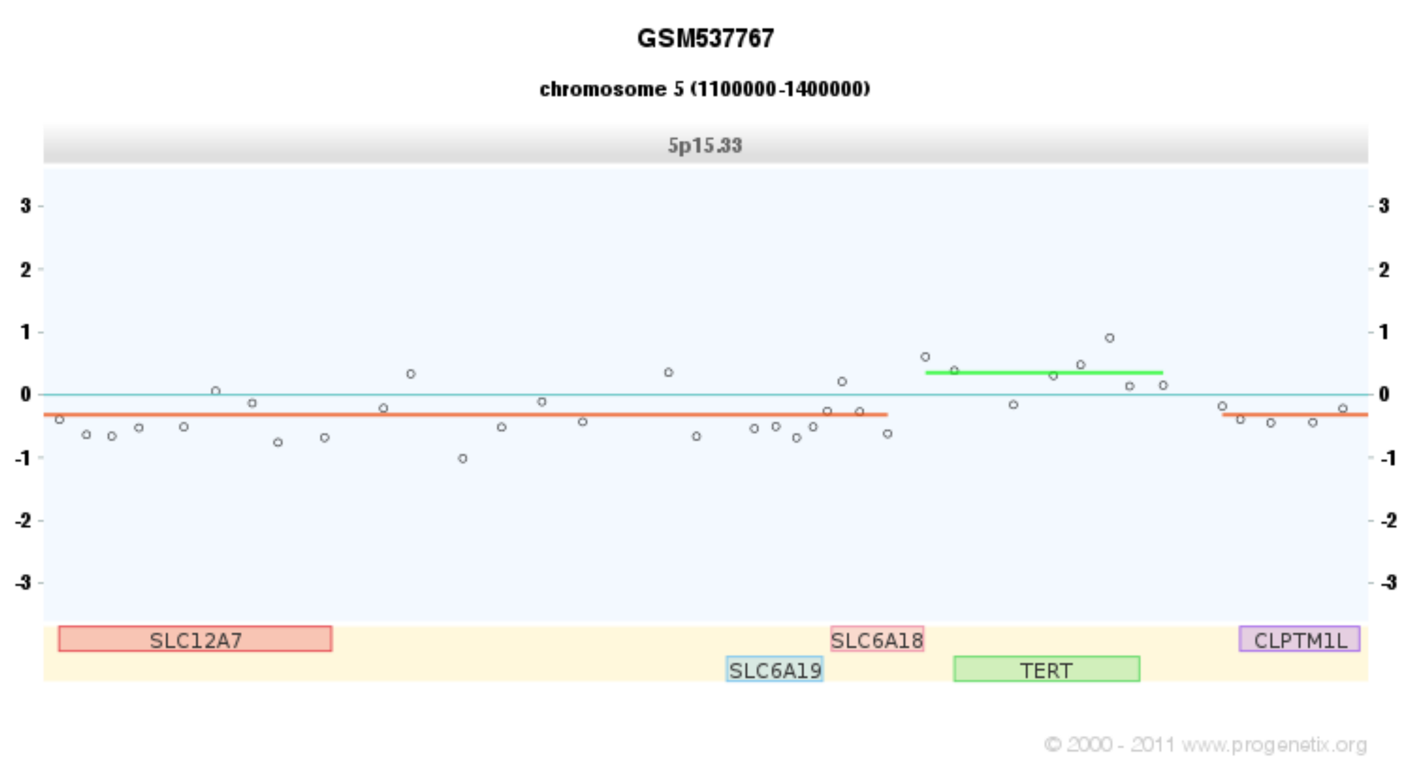

ARRAYPLOT,CHR5,1100000-1400000 PNG SVG

SELECT ALL	☑	[?]
	Visualize Data Summary	

These tools were developed for our research projects. You are welcome to try them out, but there is only sparse documentation. If more support and/or custom analysis is needed, please contact Michael Baudis regarding a collaborative project.

© 2000 - 2011 Michael Baudis, refreshed 17:25:28 (GMT), 2011-11-20. No responsibility is taken for the correctness of the data presented nor the results achieved with the Progenetix software analysis tools.
