# Supplementary material for: arrayMap: A Reference Resource for Genomic Copy Number Imbalances in Human Malignancies
Source: PLoS One. 2012 May 18;7(5):e36944. doi: 10.1371/journal.pone.0036944 (PMC3356349; doi:10.1371/journal.pone.0036944)
Supplement: Figure S4 — Compound CNA query. (A) Four gene loci associated with glioblastoma (EGFR, PTEN, ASPM and CDKN2A) were inserted into “Match (Multiple) Regions & Types” field. 303 out of 42421 arrays were returned. (B) Classification information of these 303 arrays were displayed and can be selected for the following analysis. (C) Statistical and plot parameters can be customized. Associated data was processed by online tools, and returned results included: (D) Chromosomal ideogram and (E) histogram, show frequency of copy number aberrations; (F) Matrix plot reveals the aberration pattern of selected arrays; (G) Array classification tree generated by hierarchical Ward clustering, arrays with similar frequency of CNA are part of the tree branch. (H) Heatmap of CNA frequencies clustered by clinical group. (PDF) [file pone.0036944.s004.pdf]

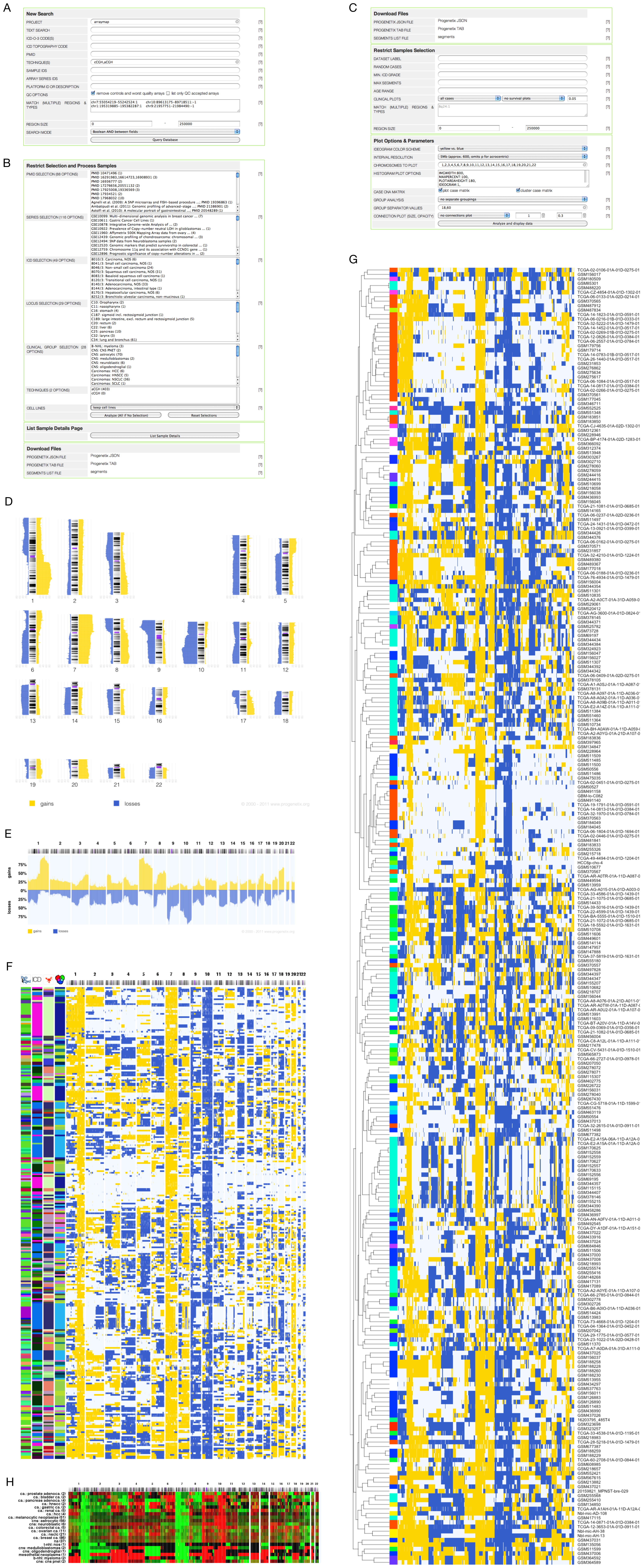
A
New Search
PROJECT
TEXT SEARCH
ICD-O-3 CODE(S)
ICD TOPOGRAPHY CODE
PMID
TECHNIQUE(S)
SAMPLE IDS
ARRAY SERIES IDS
PLATFORM ID OR DESCRIPTION
QC OPTIONS
REGION SIZE
SEARCH MODE
Query Database
B
Restrict Selection and Process Samples
List Sample Details Page
List Sample Details
Download Files
PROGENETIX JSON FILE
PROGENETIX TAB FILE
SEGMENTS LIST FILE
C
Download Files
Restrict Samples Selection
Plot Options & Parameters
Analyze and display data
D
gains
losses
E
gains
losses
F
G
H
