# Supplementary figures and images for: arrayMap: A Reference Resource for Genomic Copy Number Imbalances in Human Malignancies
Source: PLoS One. 2012 May 18;7(5):e36944. doi: 10.1371/journal.pone.0036944 (PMC3356349; doi:10.1371/journal.pone.0036944)

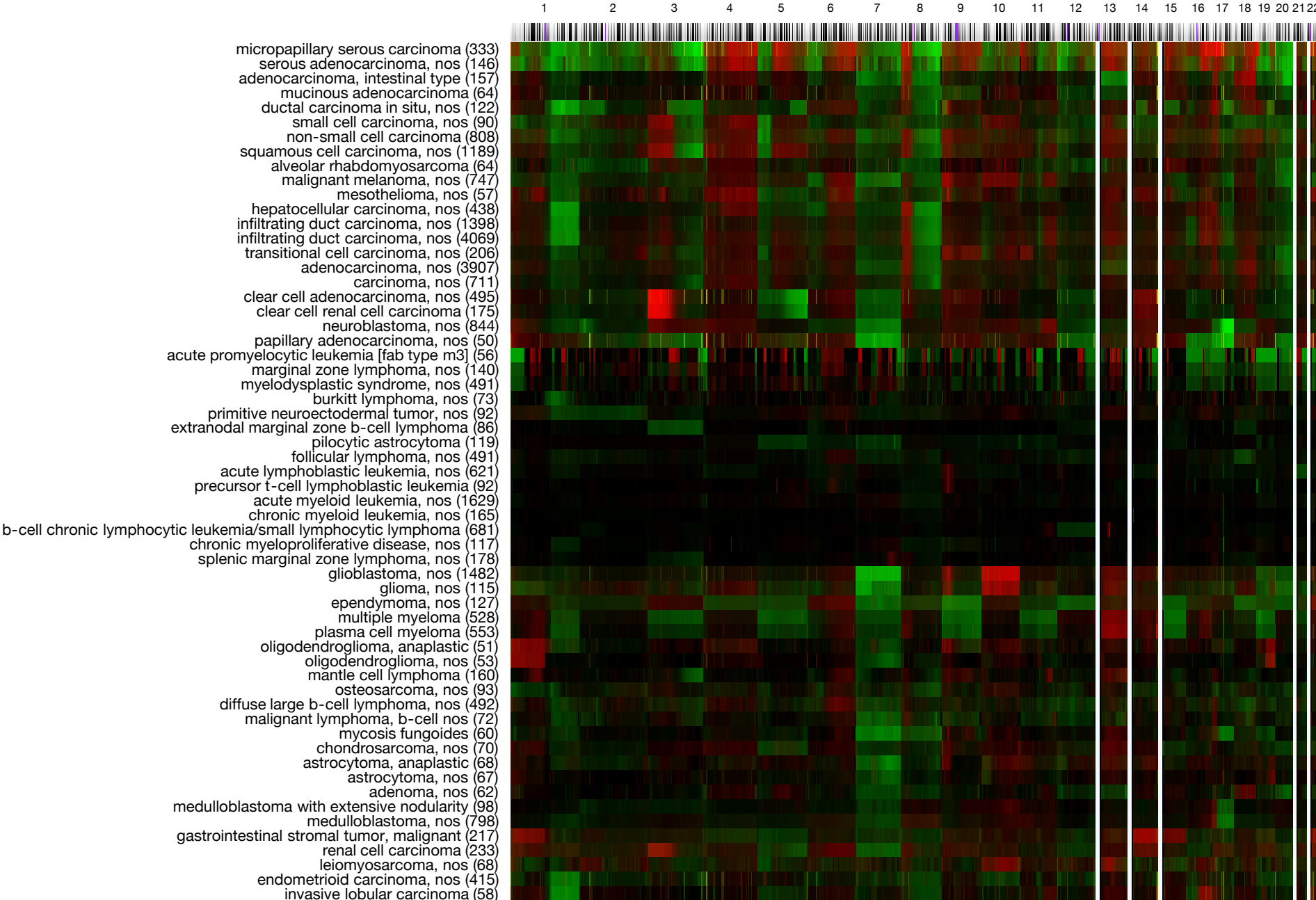

Supplement: Figure S5 — Heatmap of frequency profiles for 59 cancer types. Heatmap visualization of frequency profiles for all ICD-O entities containing more than 50 arrays in our core dataset. Region specific gain/loss frequencies were mapped to 1MB intervals. The intensity of colors (green: gains; losses: red) corresponds to the relative frequency of CNAs for each interval. (PDF) [file pone.0036944.s005.pdf]
