# Supplementary material for: arrayMap: A Reference Resource for Genomic Copy Number Imbalances in Human Malignancies
Source: PLoS One. 2012 May 18;7(5):e36944. doi: 10.1371/journal.pone.0036944 (PMC3356349; doi:10.1371/journal.pone.0036944)
Supplement: Figure S2 — Screenshot of single array visualization. ArrayMap plots for GSM630977 (acute myelogenous leukemia). Besides the whole genome view, subviews of each chromosome are displayed as well. From these plots, different kinds of genetic variation events are clearly revealed, e.g. massive genomic rearrangement in chromosome 6; arm-level gain of chromosome 8q and 3MB focal change around 1p31.3. Through the “Plot Array Data” interface, users can segment the raw data values and re-plot the results with customized parameters. (PDF) [file pone.0036944.s002.pdf]

arrayMap

Search Public Series
Search Public Arrays
Plot Array Data
Progenetix

University of Zurich[UZH]

News
Citation
FAQ & Guide
Contributing Authors
CompBio Zurich
Atlas of Cytogenetics in Hematology/Oncology
NCI SKY/M-FISH and CGH Database
NCI Cancer Chromosomes

FOLLOW US ON twitter

Share

(cc) BY-NC-ND

130.60.44.246
130.60.93.222

Visualization of genomic array data sets

SERIES	GSE23452	[?]
ARRAY ID	GSM630977	[?]
	Show original plots & Load defaults (if existing)	

CASE DATA PLOTTING	no plot refresh			[?]
GOLDEN PATH EDITION	HG18 / 36			[?]
PLOT REGION				[?]
CHROMOSOMES TO PLOT	1:22			[?]
LOSS/GAIN THRESHOLDS	-0.15 - 0.15	Y AXIS FACTOR	1.2	[?]
REGION SIZE	0 - 250000	MIN. PROBES	2	[?]

EMAIL / PASSWORD	your registered email / your received password	[?]
LICENSE AGREEMENT	I agree to license, disclaimer & citation note ☑	
	Filter / replot array data	

GSM630977	147 segments

parsing database ...

ARRAYID	GSM630977
PGCASEID	GSM630977
ARRAYNAME	MIAML041T
ARRAYDESCRIPTION	primary human: blood or bone marrow, FACS sort-purified AML blasts
DIAGNOSISTEXT	Acute Myelogenous Leukemia
ICDMORPHOLOGY	Acute myeloid leukemia, NOS
ICDMORPHOLOGYCODE	9861/3
ICDTOPOGRAPHY	hematopoietic and reticuloendothelial systems
ICDTOPOGRAPHYCODE	C42
PMID	20729466
PLATFORMID	GPL6801
PLATFORMLABEL	GenomeWideSNP_6
PROBES	1880794
QCVETO	accept
SERIESID	GSE23452
SAMPLESOURCE	primary

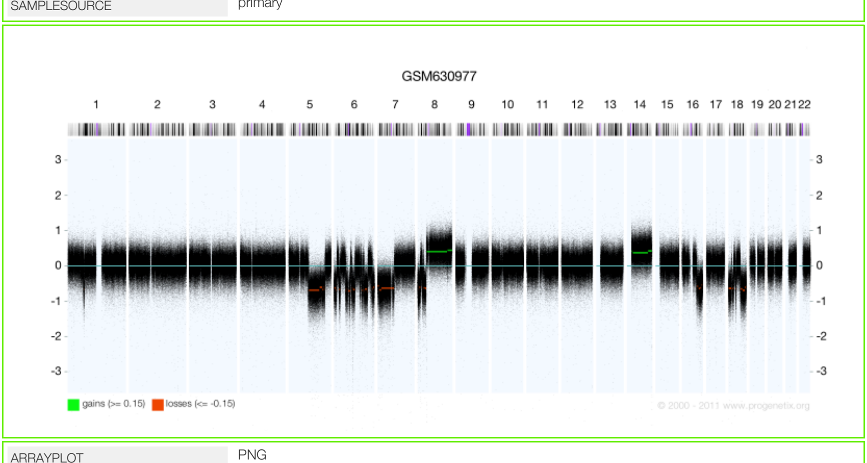

ARRAYPLOT PNG

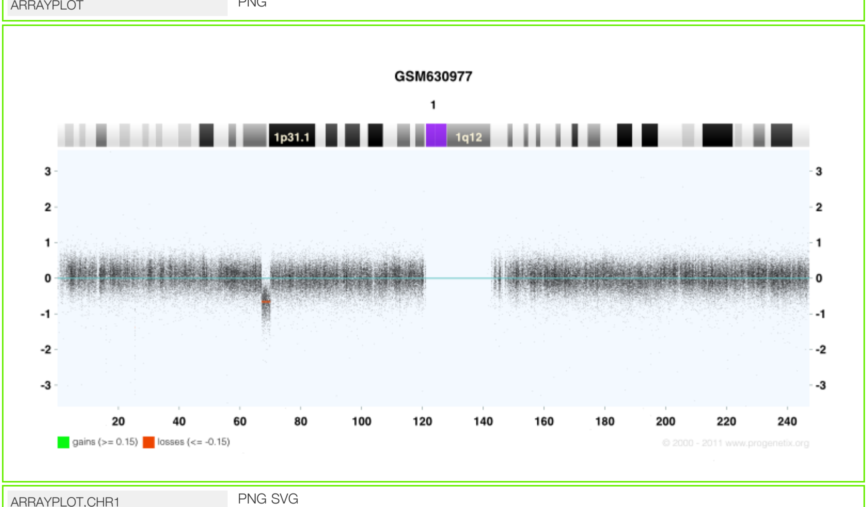

ARRAYPLOT,CHR1 PNG SVG

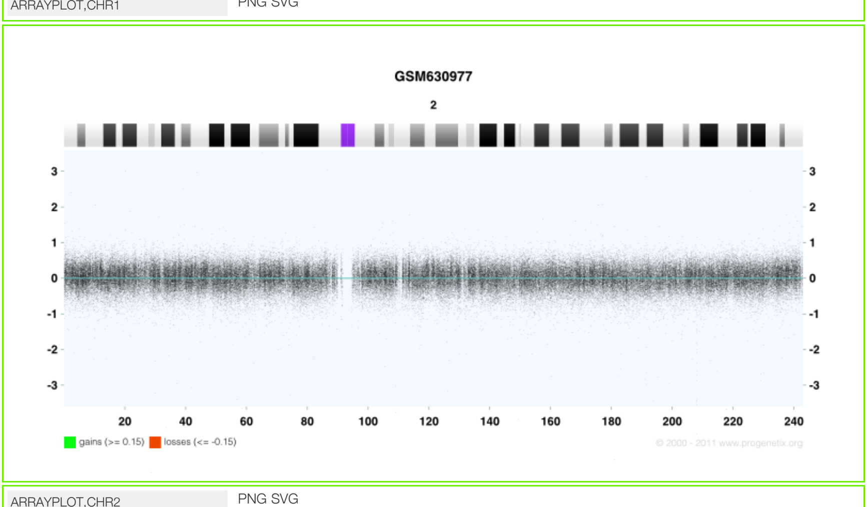

ARRAYPLOT,CHR2 PNG SVG

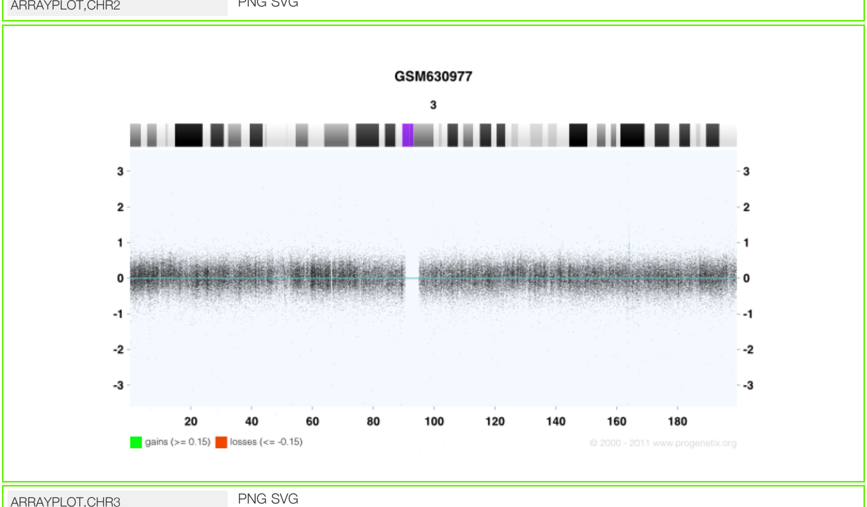

ARRAYPLOT,CHR3 PNG SVG

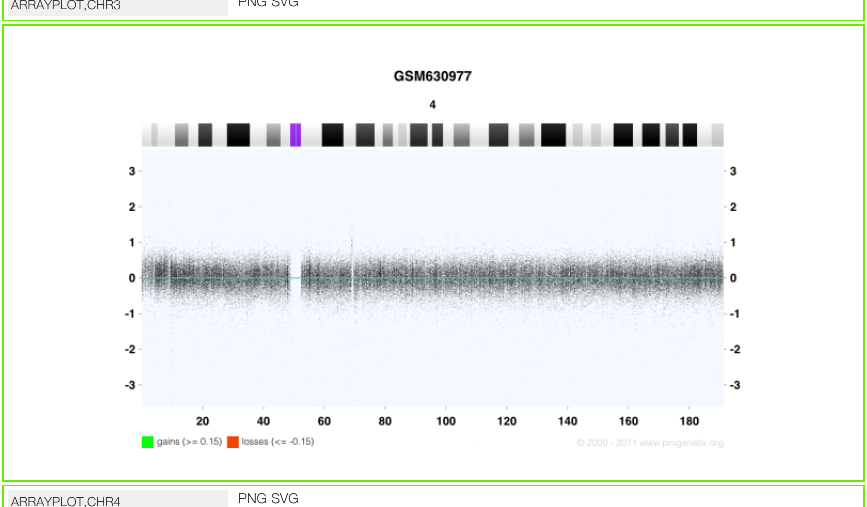

ARRAYPLOT,CHR4 PNG SVG

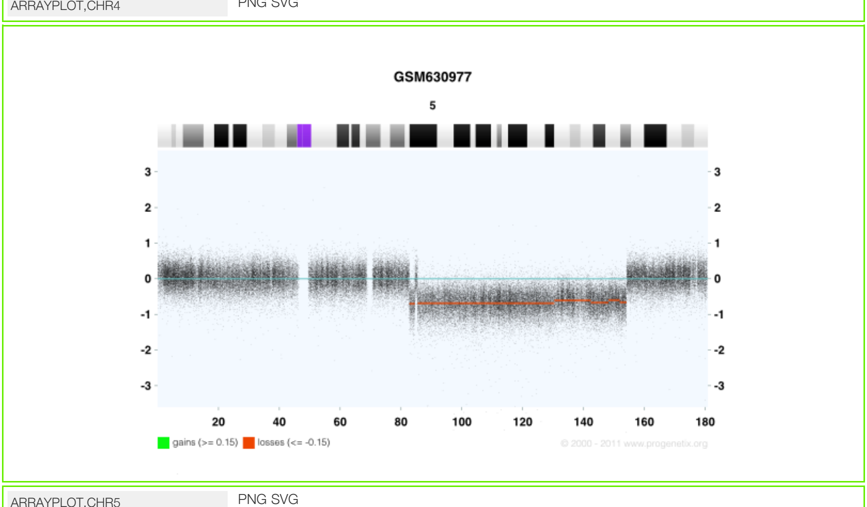

ARRAYPLOT,CHR5 PNG SVG

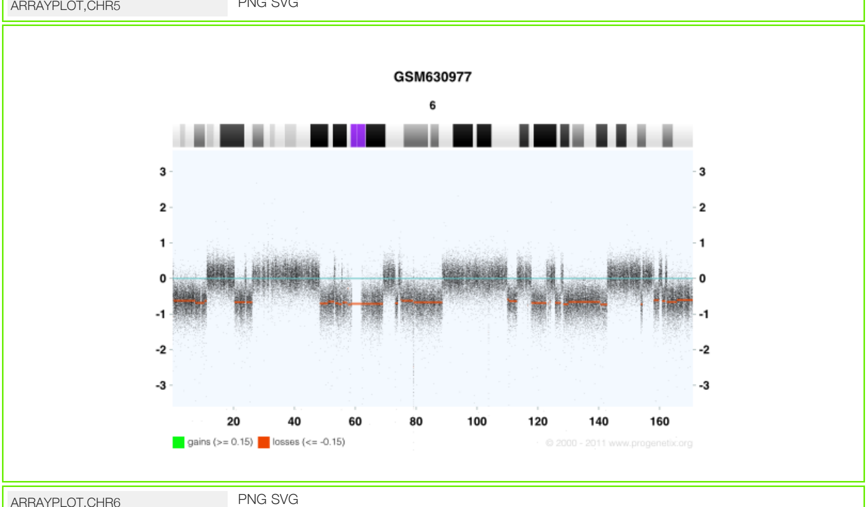

ARRAYPLOT,CHR6 PNG SVG

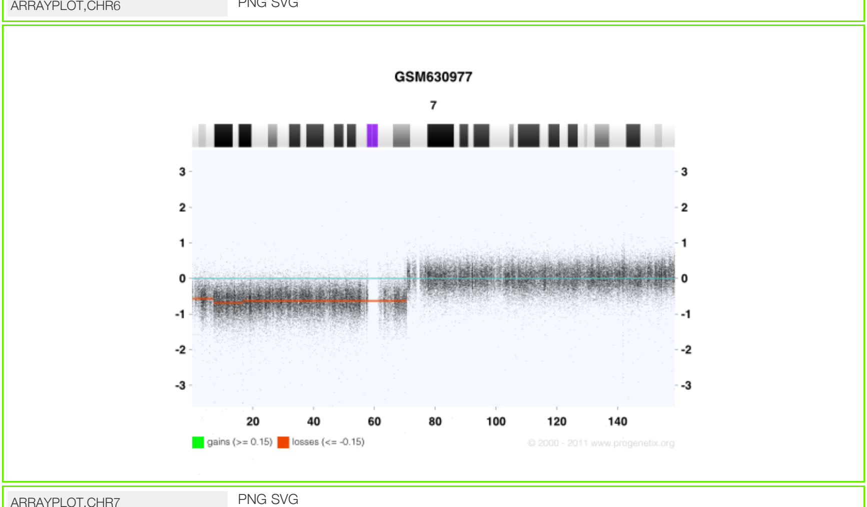

ARRAYPLOT,CHR7 PNG SVG

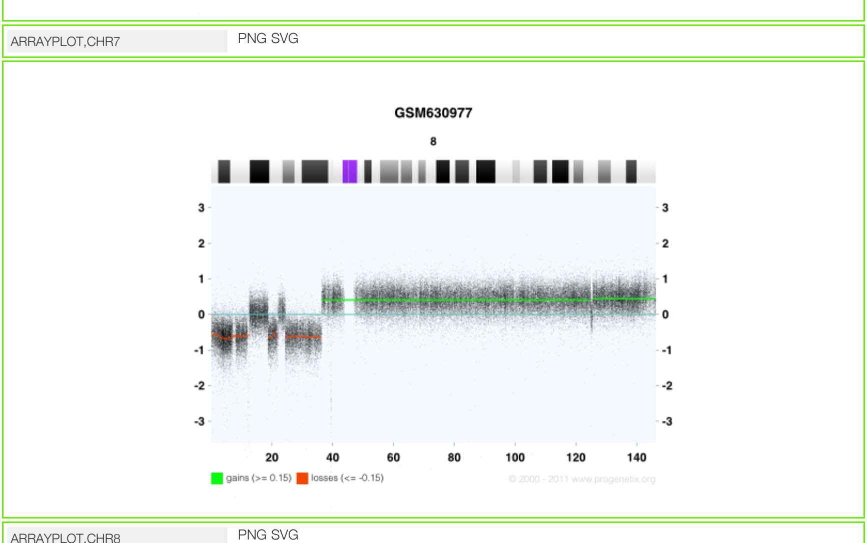

ARRAYPLOT,CHR8 PNG SVG

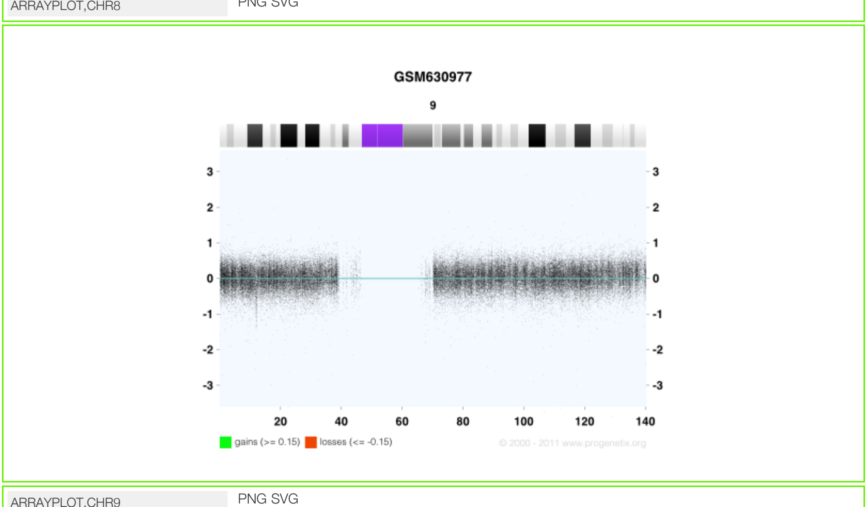

ARRAYPLOT,CHR9 PNG SVG

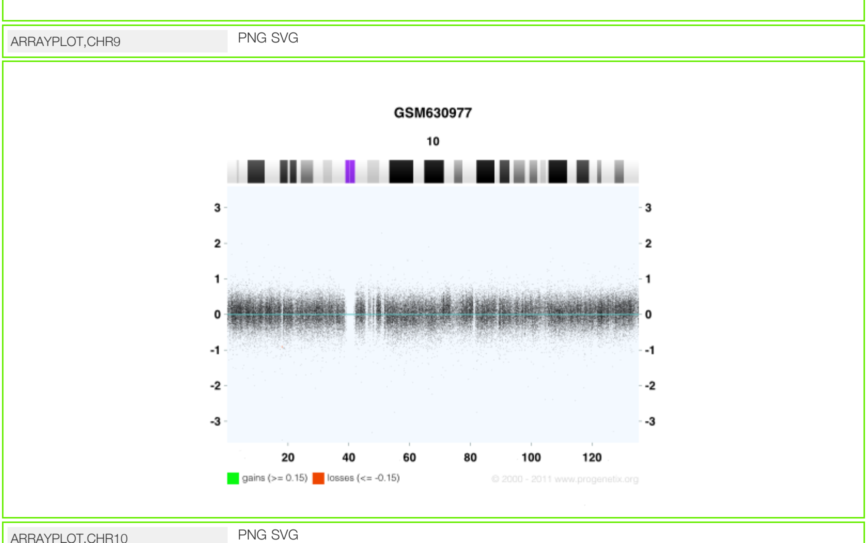

ARRAYPLOT,CHR10 PNG SVG

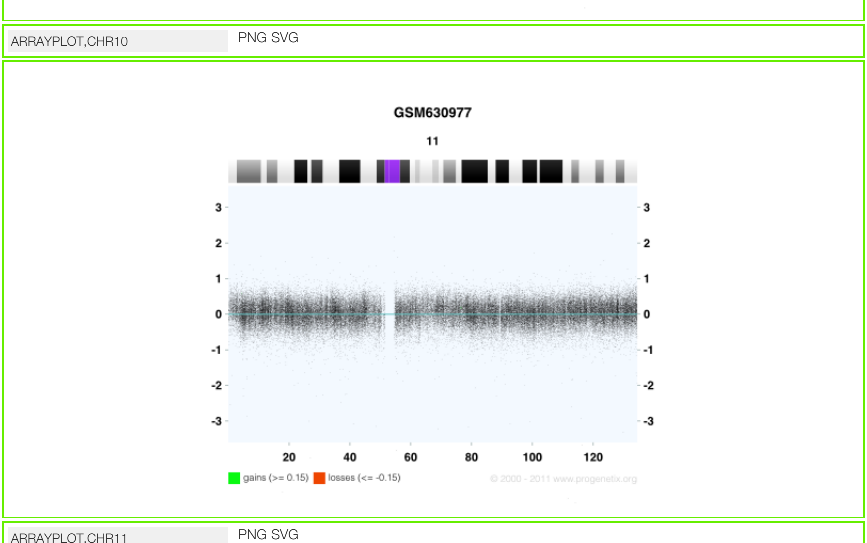

ARRAYPLOT,CHR11 PNG SVG

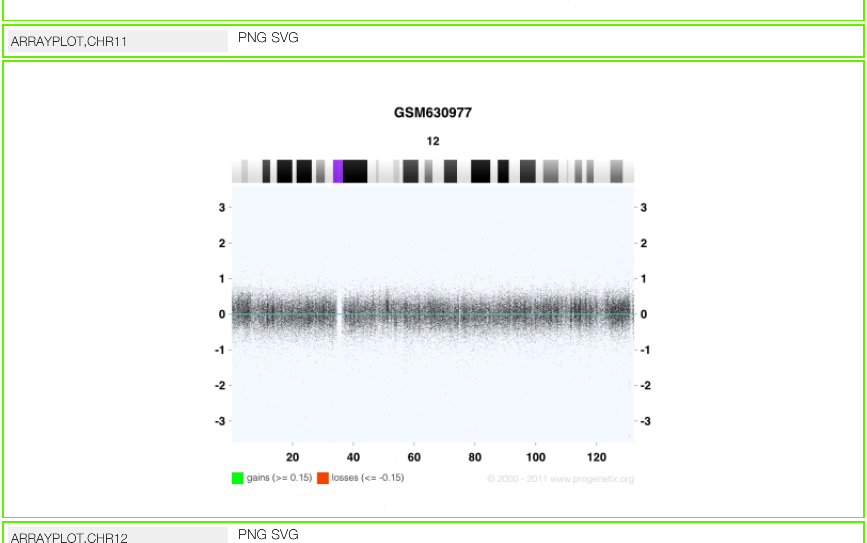

ARRAYPLOT,CHR12 PNG SVG

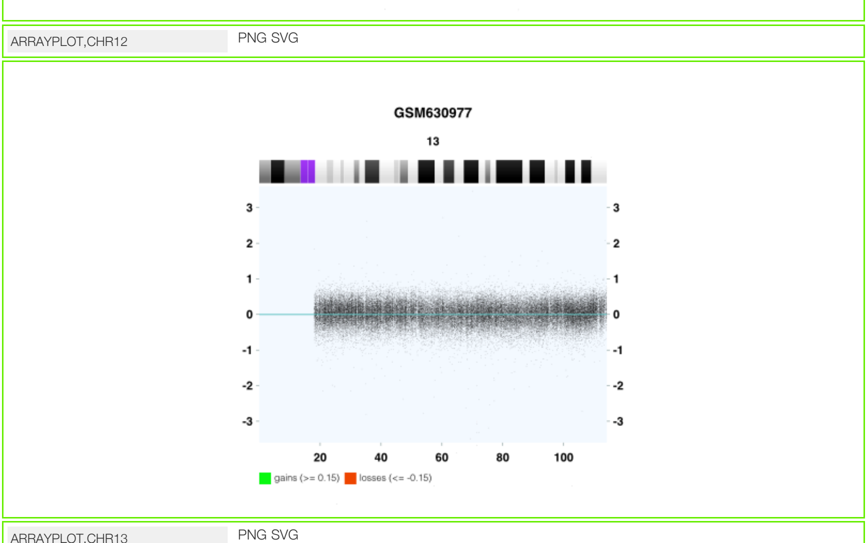

ARRAYPLOT,CHR13 PNG SVG

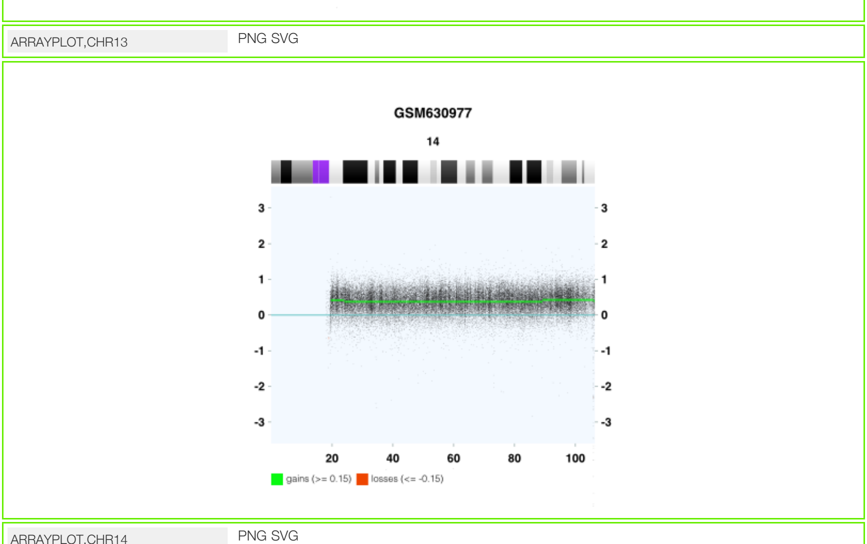

ARRAYPLOT,CHR14 PNG SVG

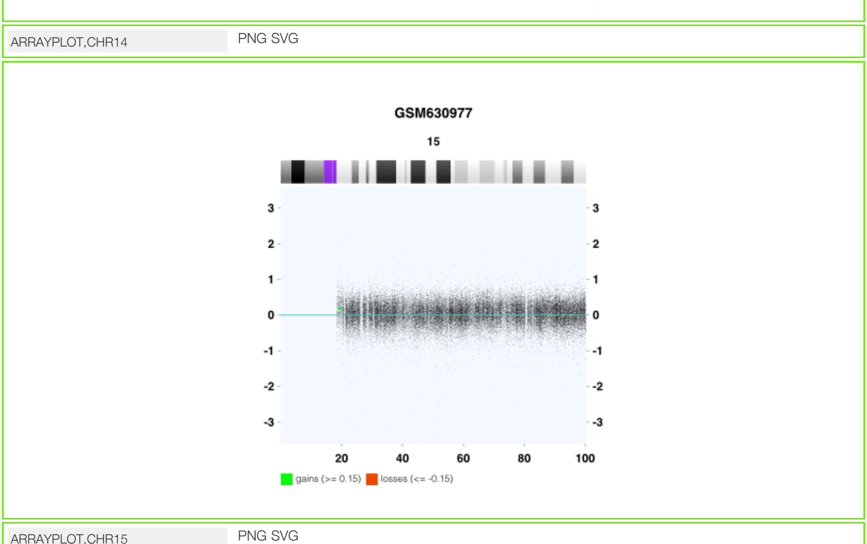

ARRAYPLOT,CHR15 PNG SVG

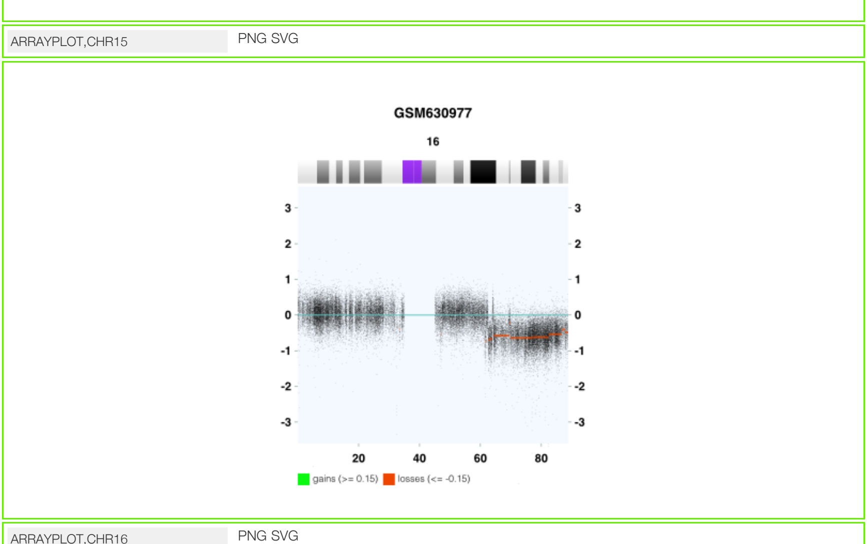

ARRAYPLOT,CHR16 PNG SVG

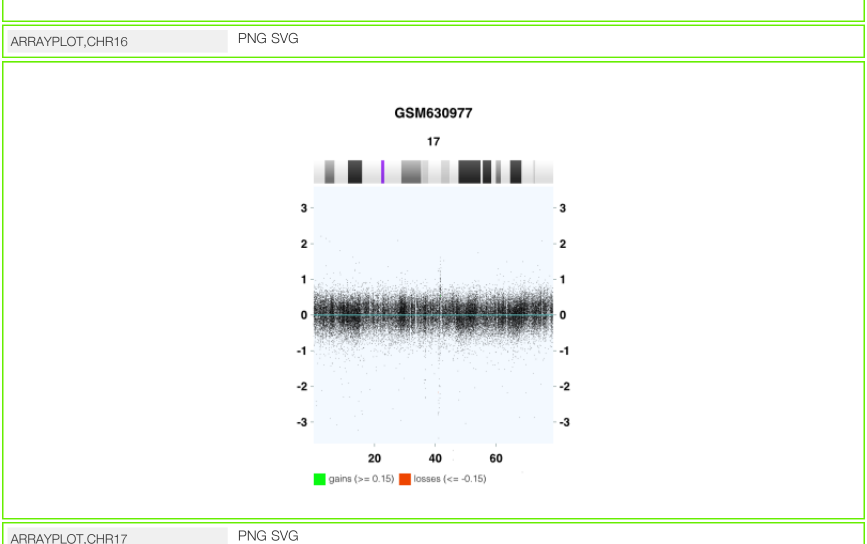

ARRAYPLOT,CHR17 PNG SVG

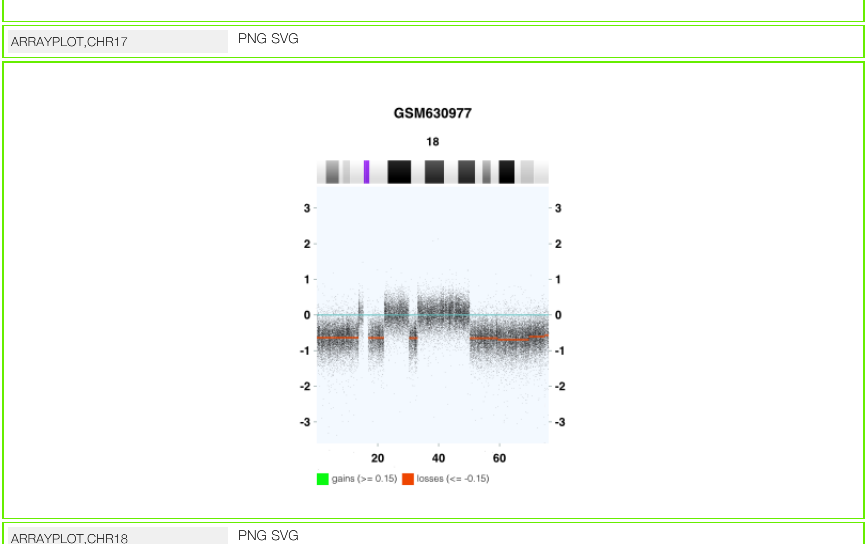

ARRAYPLOT,CHR18 PNG SVG

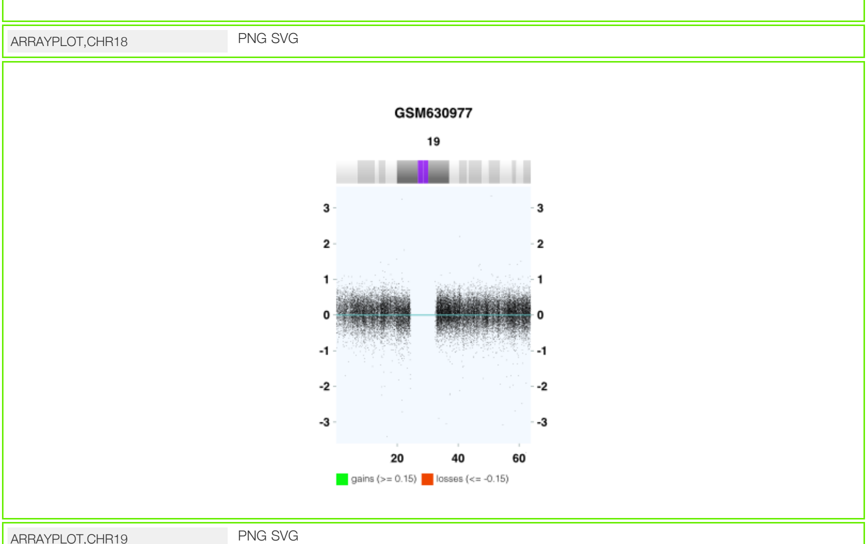

ARRAYPLOT,CHR19 PNG SVG

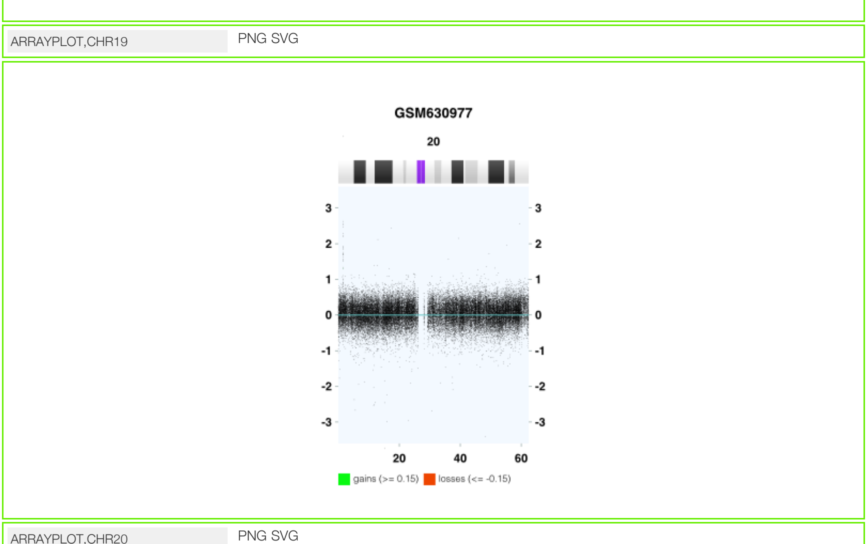

ARRAYPLOT,CHR20 PNG SVG

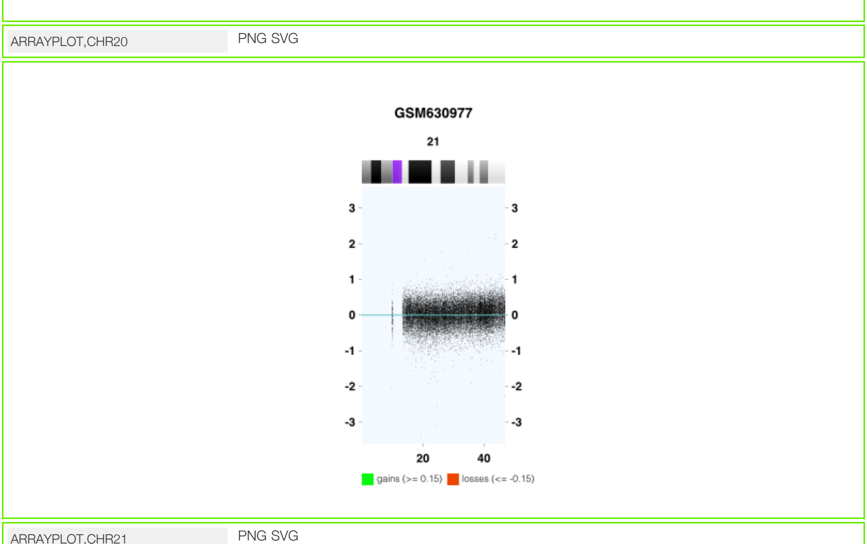

ARRAYPLOT,CHR21 PNG SVG

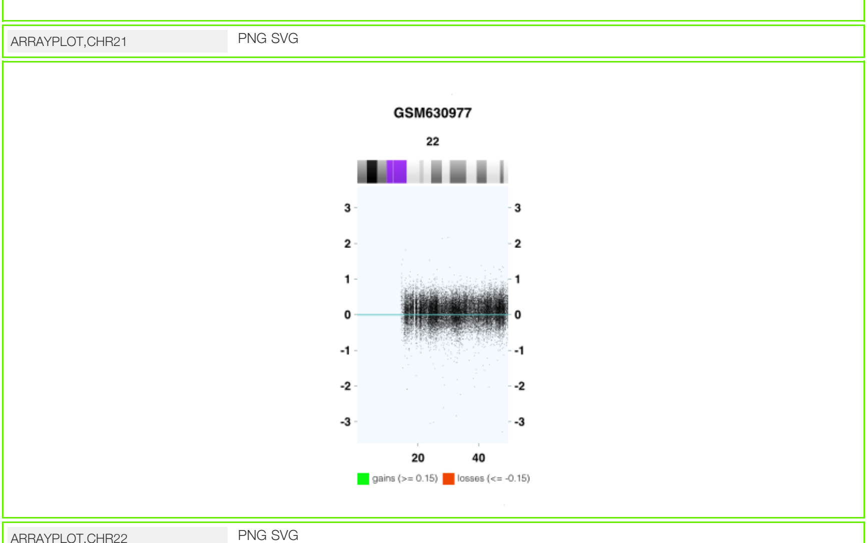

ARRAYPLOT,CHR22 PNG SVG

SELECT ALL	☑	[?]
	Visualize Data Summary	
